# Spontaneous Pneumomediastinum, Pneumoperitoneum, and Pneumoretroperitoneum After Endoscopic Cryoablation Without Frank Perforation

**DOI:** 10.14309/crj.0000000000000204

**Published:** 2019-08-14

**Authors:** Brendan Chen, Jason Ferreira

**Affiliations:** 1 Department of Medicine, Alpert Medical School of Brown University, Providence, RI; 2 Division of Gastroenterology, Alpert Medical School of Brown University, Providence, RI

## Abstract

Perforation after endoscopic cryoablation is a rare but serious complication. We present a middle-aged male patient who presented for an elective session of endoscopic cryoablation for his Barrett esophagus with high-grade dysplasia. After cryoablation, the patient complained of abdominal pain, and his abdomen became distended and tympanic. Computed tomography showed pneumomediastinum, pneumoperitoneum, and pneumoretroperitoneum but no evidence of extraluminal contrast extravasation. The patient was treated with antibiotics and had no complications. To our knowledge, this is the first described case of pneumomediastinum, pneumoperitoneum, and pneumoretroperitoneum without frank perforation after endoscopic cryoablation.

## INTRODUCTION

Management of Barrett esophagus with high-grade dysplasia requires endoscopic eradication therapy. The most common modality is by radiofrequency ablation, although cryoablation has become popular as an alternative.^1,2^ Cryoablation involves freezing and subsequently destroying dysplastic cells using liquid nitrogen, carbon dioxide, or nitrous oxide. Currently, the most studied and most popular cryogen used is liquid nitrogen, which has shown good curative rates with few complications. Complications can range from pain and bleeding to strictures and rarely perforation.^3–7^ On imaging, esophageal perforation is often marked by abnormal air in surrounding structures such as the mediastinum or peritoneum.

## CASE REPORT

**Figure 1.**
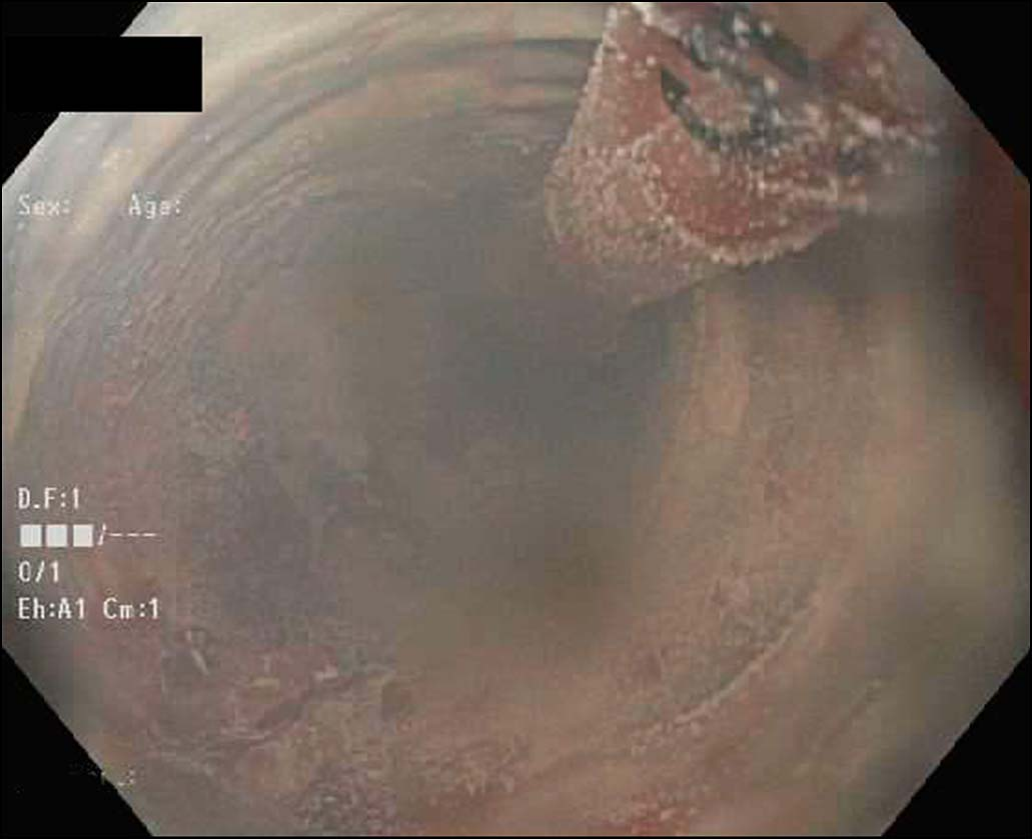
Liquid nitrogen cryoablation of the patient's dysplastic esophagus.

**Figure 2.**
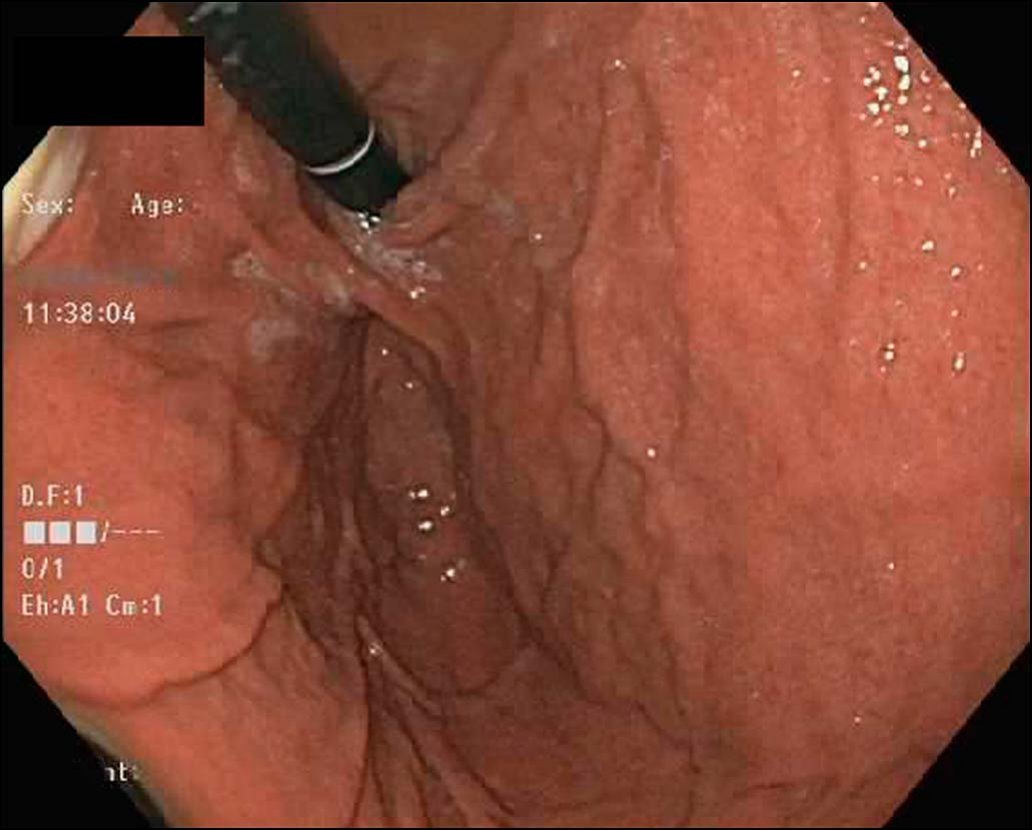
Retroflexion of the stomach showing no indication of gastric wall defects.

**Figure 3.**
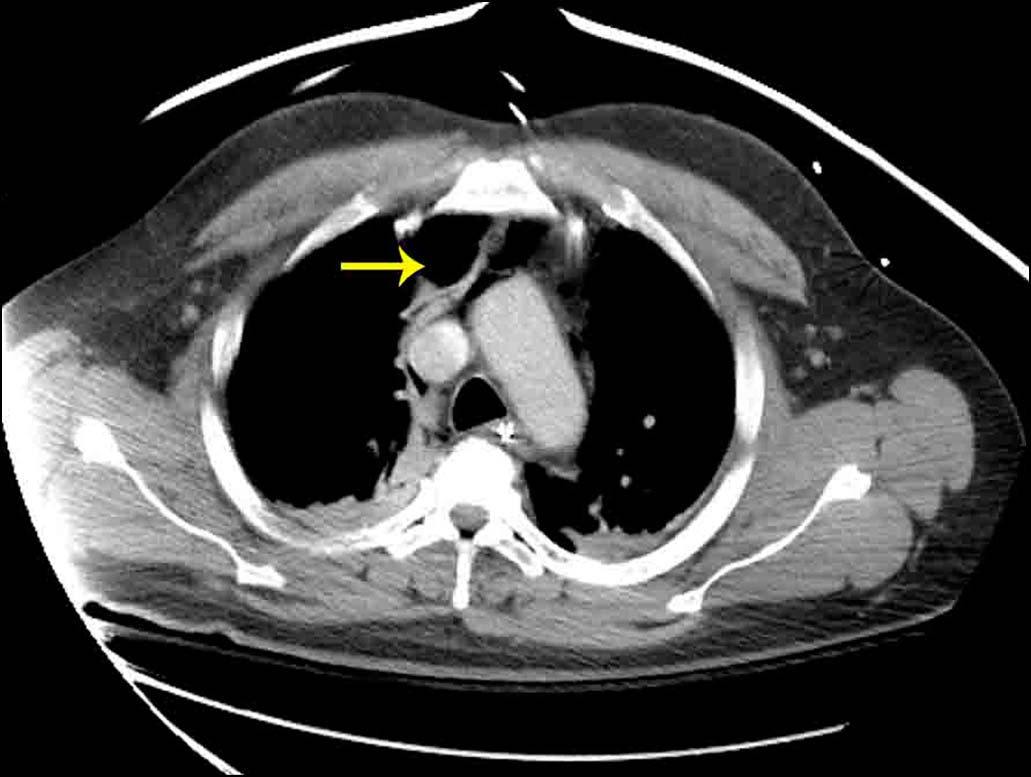
CT showing pneumomediastinum (arrow) following cryoablation. CT, computed tomography.

**Figure 4.**
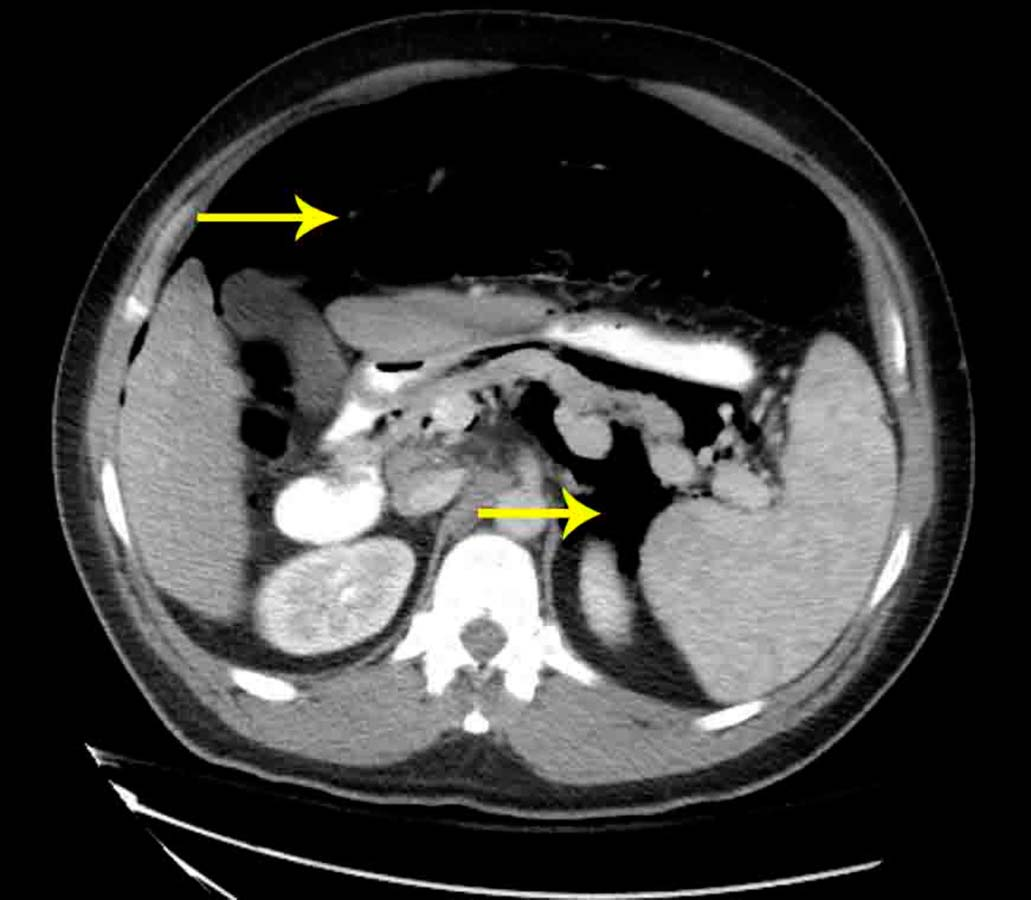
CT showing pneumoperitoneum (top arrow) and pneumoretroperitoneum (bottom arrow) following cryoablation. CT, computed tomography.

**Figure 5.**
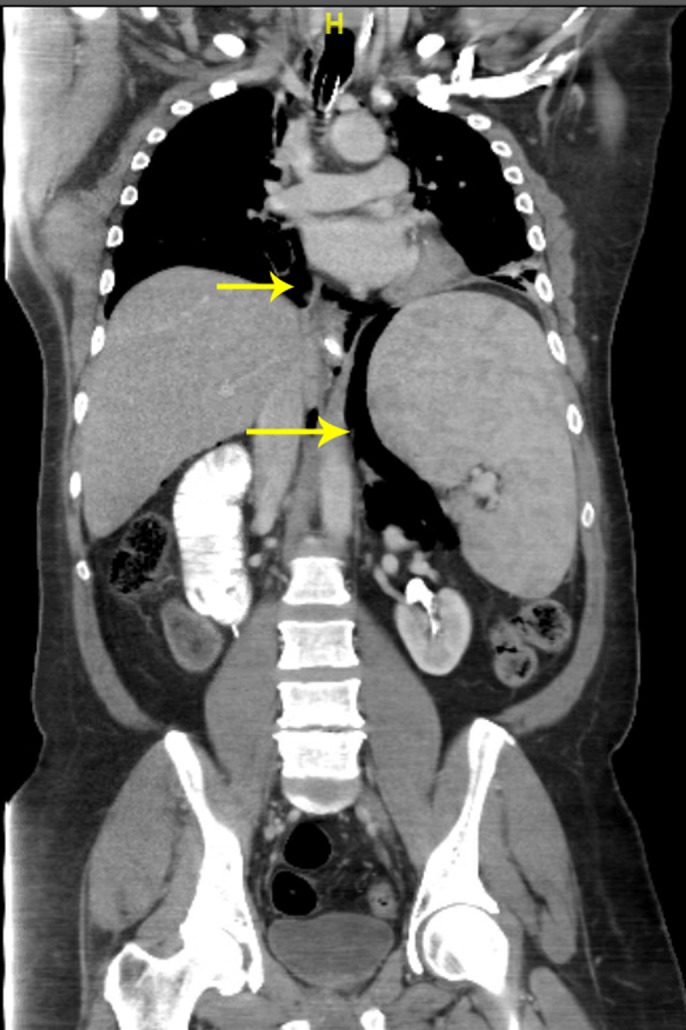
CT showing pneumomediastinum (top arrow), pneumoretroperitoneum (bottom arrow), following cryoablation. CT, computed tomography.

A 54-year-old man with a history of Hepatitis C and compensated cirrhosis presented for an elective second session of endoscopic cryoablation for his Barrett esophagus with high-grade dysplasia. After undergoing 3 cryoablation cycles with liquid nitrogen, the patient's abdomen became distended and tympanic (Figure 1). He was noted to be taking shallow breaths. A 16-gauge Angiocath was inserted into his abdominal cavity above the umbilicus, and air was expressed from his peritoneal cavity indicative of a perforation. Repeat esophagogastroduodenoscopy was performed to try to find the area of perforation, so endoscopic treatment could be pursued. However, his repeat esophagogastroduodenoscopy was normal, and his duodenum, stomach, and esophagus insufflated well with no apparent leakage and no obvious mucosal defect was seen to indicate perforation (Figure 2). The Angiocath was removed, and his abdomen remained soft. When he was awakened from sedation, he was complaining of severe abdominal pain and in respiratory distress prompting repeat intubation. Computed tomography showed pneumomediastinum, pneumoperitoneum, and pneumoretroperitoneum with no evidence of extraluminal contrast extravasation (Figures 3–5). The patient was empirically started on piperacillin-tazobactam and fluconazole for perforation. The patient had a barium swallow, which was also negative for perforation. The patient did well and was extubated the following day and discharged 2 days later with a short course of amoxicillin-clavulanate.

## DISCUSSION

Endoscopic cryoablation has been shown to be a safe therapy for Barrett esophagus with high-grade dysplasia.^3–5^ Perforations following cryoablation are rare and have only been reported 3 times in the literature (Table 1).^7–9^

**Table 1.**
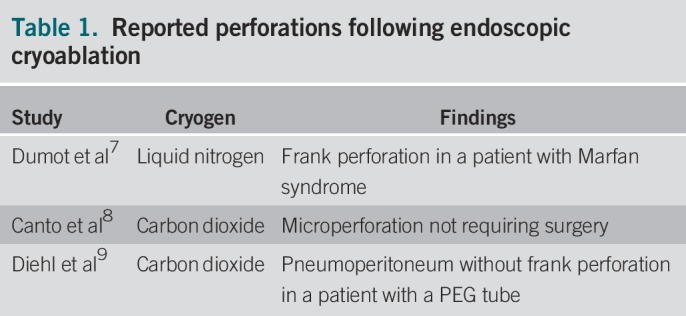
Reported perforations following endoscopic cryoablation

To our knowledge, this is the first described case of pneumomediastinum, pneumoperitoneum, and pneumoretroperitoneum without frank perforation after endoscopic cryoablation. In our patient, it was likely secondary due to barotrauma on the gastrointestinal mucosa from the liquid nitrogen despite adequate ventilation through an orogastric tube to suction during the procedure. There has been one other case of pneumoperitoneum without frank perforation, but in that report, carbon dioxide was used, and the patient also had a percutaneous endoscopic gastrostomy tube, which may have weakened the peritoneum's ability to withstand barotrauma.^9^ In our patient, he did not have any known structural defects, and it is unlikely that his cirrhosis could have been a risk factor.

In our patient, prompt recognition of possible perforation and rapid decompression of the abdomen cryoablation may have been life-saving. For suspected perforation, we recommend using carbon dioxide insufflation and rapid Angiocath placement as we did in our case.

## DISCLOSURES

Author contributions: All authors contributed equally to the manuscript. B. Chen is the article guarantor.

Financial disclosure: None to report.

Informed consent was obtained for this case report.
